# Autoantibodies against the Second Extracellular Loop of M3R Do neither Induce nor Indicate Primary Sjögren’s Syndrome

**DOI:** 10.1371/journal.pone.0149485

**Published:** 2016-02-22

**Authors:** Yan Chen, Junfeng Zheng, Qiaoniang Huang, Fengyuan Deng, Renliang Huang, Wenjie Zhao, Junping Yin, Lina Song, Juan Chen, Xing Gao, Zuguo Liu, Frank Petersen, Xinhua Yu

**Affiliations:** 1 Laboratory of Autoimmunity, Medical College of Xiamen University, Xiamen, China; 2 Department of Pharmacy, The First Affiliated Hospital of Xinxiang Medical University, Henan, China; 3 Department of Rheumatology of the First Afflict Hospital of Xiamen University, Xiamen, China; 4 Department of Clinical laboratory, Xiamen University Hospital, Xiamen University, Xiamen, China; 5 Eye Institute and Affiliated Xiamen Eye Center of Xiamen University, Xiamen, China; 6 Priority Area Asthma & Allergy, Research Center Borstel, Airway Research Center North (ARCN), Members of the German Center for Lung Research (DZL), Borstel, Germany; National Institute of Dental and Craniofacial Research, UNITED STATES

## Abstract

**Objectives:**

Anti-muscarinic acetylcholine type-3 receptor (anti-M3R) autoantibodies have been suggested to be pathogenic for primary Sjögren’s syndrome (pSS), and the second extracellular loop of M3R is suspected to carry a disease-promoting epitope. In this study, we aimed to evaluate the pathogenicity of autoantibodies against peptides derived from the second extracellular loop of M3R in mice and to determine whether those autoantibodies could be used as biomarker for pSS.

**Methods:**

BALB/c mice were immunized with modified linear or cyclic peptides of the second extracellular loop of M3R. The function of exocrine glands was evaluated by measuring the secretion of saliva and tears. The histological evaluations were performed by using H&E staining or direct immunofluorescence staining. Autoantibodies against linear or cyclic peptides of the second extracellular loop of M3R in human and mice were determined using ELISA.

**Results:**

Immunization induced mice to produce autoantibodies against the linear or cyclic peptides of the second extracellular loop of M3R, and those autoantibodies could bind onto salivary glands. However, those mice showed neither impairment in the secretion of tears or saliva nor histological abnormality in the exocrine glands. Furthermore, passive transfer of the IgG isolated from the immunized mice into healthy mice did not induced the dysfunction of the exocrine glands. The prevalence of autoantibodies against the peptides of the second extracellular loop of M3R was low in pSS patients, and it did not differ significantly from that in healthy controls.

**Conclusions:**

Our results suggest that the autoantibodies against peptides of the second extracellular loop of M3R are not pathogenic *in vivo* and they are not suitable as biomarkers for pSS diagnosis.

## Introduction

Sjögren’s syndrome (SS) is a chronic autoimmune disease targeting the exocrine glands and leading to the dry eyes (xerophthalmia) and dry mouth (xerostomia) [1]. It affects 0.4–4% of the general population, with a female to male ratio reaching 9:1 [2,3]. This disease can develop alone as primary SS (pSS), while it occurring with other autoimmune diseases such as rheumatoid arthritis or systemic lupus erythematosus is called secondary Sjögren's syndrome (2ndSS). The pSS is characterized by a panel of circulating autoantibodies including anti-SSA, anti-SSB, anti-muscarinic type 3 acetylcholine receptors (M3R), and anti-α fodrin antibodies [4]. However, pathogenic autoantibodies for pSS have not been identified so far.

M3R is expressed on many tissue including exocrine glands and has an important role in exocrine secretion[5,6]. Autoantibodies against M3R have been detected in majority of patients with pSS [7] as well as in NOD/Lt mice which develop spontaneous pSS-like disease [8]. An *in vitro* study has demonstrated that autoantibodies against M3Rare able to inhibit the secretion function of the human submandibular salivary acinar cells [9]. Furthermore, evidence from mouse studies also support a role of M3R in the development of pSS. In 2010, Iizuka and colleagues established a mouse model of pSS by immunizing M3R-/- mice with M3R peptides and then transferring their splenocytes to Rag2-/- mice[10], showing a pathogenic role of immune response against M3R in pSS. Taken together, these evidences indicate that M3Rcould represent one putative candidate for a pathogenic autoantigen in pSS.

M3R, a G-coupled protein receptor contains four extracellular domains including N-terminal and three extracellular loops [5]. A random mutagenesis study has demonstrated that some amino acid residues within the second extracellular loop of M3R are critical for the agonists mediated receptor activation[11], suggesting that the second extracellular loop may serve as agonist binding site. Given the important role of the second extracellular loop in the function of M3R, many efforts have been made to identify a potential pathogenic epitope for pSS in this domain. In 2004, Cavill et al. reported that rabbit polyclonal antibodies raised against a synthetic peptide derived from the second extracellular loop of human M3R could inhibit the receptor function *in vitro* [12]. The inhibitory role of the antibodies against this peptide was confirmed by Tsuboi and his colleagues who reported that monoclonal antibodies raised against this peptide can block the M3R mediated activation in a human salivary gland cell line [13]. Furthermore, He et al. reported that the autoantibodies against the peptides of the second extracellular loop of M3R could be detected in pSS patients and that the presence of those autoantibodies were correlated with the salivary flow rate and disease severity in patients [14,15]. Although these studies provide indirect evidence for a pathogenic role of autoantibodies against the peptide of the second extracellular loop of M3R, a direct proof for this *in vivo* is still missing.

Beside their pathophysiological relevance, anti-M3R autoantibodies have also been extensively evaluated as novel biomarker for pSS diagnosis. Using a cell line expressing M3R, Gao *et al* could detectanti-M3R autoantibodies in the sera of the majority of pSS patients [7]. Since a cellline-based method is not applicable to the clinical diagnosis, many investigators have tried to detect those autoantibodies against M3R using peptide-based ELISA systems [16,17]. Particularly, He *et al* reported that autoantibodies against a cyclic peptide of the second extracellular loop of M3R could serve as a useful biomarker for pSS [14]. However, these promising results have not been confirmed by other groups [18–20], and the suitability of such peptide-based ELISA as a diagnosis tool for detecting anti-M3R autoantibodies in pSS remains unclear.

In this study, we investigated the relevance of autoantibodies against peptides of the second extracellular loop of M3R in pSS. The potential pro-pathogenic role of these autoantibodies was studied in animal models in mice *in vivo*. Furthermore, a peptide-based M3R ELISA was used to evaluate anti-M3R autoantibodies as potential biomarkers for pSS in patients.

## Materials and Methods

### Patients and healthy control subjects

Fifty two pSS patients (45 females and 7 males) were enrolled at the department of rheumatology, the First Afflict Hospital of Xiamen University. All patients were diagnosed according to the standards defined by criteria of the American–European Consensus Group in 2002 [21]. The age of the 52 pSS patients ranged from 24 to 71 years (mean age of 44.3 years). Fifty six age-and gender-matched healthy controls were recruited from the Xiamen University Hospital. All serum samples were prepared freshly from peripheral blood samples of patients and controls and stored at -80°C until use. Approval for these studies was obtained from the institutional ethics committee of Xiamen University according to the Declaration of Helsinki. All volunteers gave written informed consent.

### Peptides

The peptides used in this study are listed in the Table 1. This includes the peptides of the murine M3R extracellular N-terminus domain (N_ter_), the 1stEL of murine M3R (1stEL), the 2ndEL of murine/human M3R (2ndEL), the cyclic peptide of the 2ndEL of murine/human M3R (c2ndEL), a 25-mer peptide SGSG control peptide, OVA_323–339 peptide (OVA), the OVA_323–339 conjugated 2ndECL (OVA-2ndEL), the OVA_323–339 conjugated c2ndEL (OVA-c2ndEL), biotin-labeled 2ndEL (bio-2ndEL), and biotin labeled c2ndEL (bio-c2ndEL). All peptides were synthesized at the Research Center Borstel, Germany.

**Table 1:** Peptides used in this study.

Names	Amino acid sequences of peptides
**Nter**	VHSPSEAGLPLGTVSQLDSYNISG
**1stEL**	FTTYIIMNRWALGNLACDLW
**2ndEL**	LFWQYFVGKRTVPPGWCFIQFLSEPT
c2ndEL*	CLFWQYYFVGKRTVPPGWCFIQFLSEPT
**SGSG**	SGSGSGSGSGSGSGSGSGSGSGSGSG
**OVA_323–339**	ISQAVHAAHAEINEAGR
**OVA-2ndEL**	ISQAVHAAHAEINEAGRLFWQYFVGKRTVPPGWCFIQFLSEPT
OVA-c2ndEL*	ISQAVHAAHAEINEAGRCLFWQYYFVGKRTVPPGWCFIQFLSEPT
**Biotin-2ndEL**	LFWQYFVGKRTVPPGWCFIQFLSEPT-biotin
Biotin-c2ndEL*	CLFWQYYFVGKRTVPPGWCFIQFLSEPT-biotin

Note:

*There are disulfide bonds in c2ndEL, OVA-c2ndEL and Biotin-c2ndEL peptides.

### Mice and immunization

All Balb/c mice used in this study were female and obtained from Shanghai SLAC Laboratory Animal Co. (Shanghai, China) and were housed under specified pathogen free conditions at the animal facility at the Xiamen University. All procedures and assays were approved by the Institutional Animal Care and Use Committee of Xiamen University. To induce autoantibodies against ofM3R, 8–12 weeks old female Balb/c mice were immunized at the base of the tail subcutaneously with a mixture of 100 μg peptide (OVA-2ndEL, OVA-c2ndEL or OVA) dissolved in 100 μl PBS and an equal volume of Complete Freund Adjuvant containing 4mg/ml *M*. *tuberculosis* (DIFCO, USA). Mice were boosted with a mixture of 100 μg peptide dissolved in 100 μl PBS and an equal volume of IFA 28 days after the first immunization.

### Measurement of saliva and tears

Mouse saliva and tears were collected according to a protocol published previously [22], with modifications. Briefly, mice were fasted for 12–14 hours before measurement. Chloral hydrate (350mg/kg) was applied by intraperitoneal (i.p.) injection to each mouse for anesthesia. Saliva and tears secretion was then stimulated by injection of pilocarpine (0.5mg/kg body weight, Sigma) i.p.. Directly thereafter, saliva was collected from the oral cavity for over a 20-min period using a sponge. Tears were collected at 10 min and 20 min after the pilocarpine injection using a 5μl Hirschmann microcapillary pipette (SIGMA-ALDRICH, Germany). Weights of saliva and tear volumes were normalized by body weights for the further analysis.

### Histological evaluation

Salivary and lacrimal glands were collected from sacrificed mice and stored in 4% PFA solution. The 5-μm thick sections prepared from salivary and lacrimal glands were stained with Mayer’s hematoxylin and eosin (H&E).

### IgG isolation and transfer

To isolate murine IgG, serum samples prepared from immunized mice were diluted with an equal amount of PBS (pH 7.4) and passed through a sterile filter of 0.22 μm pore size (Millipore, Ireland). Preparations were subsequently passed through a HiTrap protein G Affinity column (GE Healthcare, USA) and IgG fractions were eluted with 0.1M glycine HCl buffer (pH 2.7). Protein contents were determined at a wavelength of 280 nm in a Biophotometer (Eppendorf, Germany) and sterile IgG fractions were adjusted to 1mg/ml before storage at -80°C.

In some experiments, IgG prepared from immunized mice was transferred into naive female Balb/c mice according to a protocol published previously [23], with modification. Briefly, sterile IgG was transferred into naive 8- to 10-week-old female Balb/c mice (400 μg IgG /20g bodyweight) by a single i.p. injection. Thereafter, mice were followed for seven days post antibody transfer.

### Immunofluorescence staining

Antibody binding to salivary glands was detected using direct immunofluorescence staining. Cryosections of murine salivary glands were blocked using 3% BSA in PBS for 45 minutes at room temperature and incubated with D649-goat-anti-mouse IgG antibodies (Biolegend, USA). After washing, sections were stained with DAPI (Solarbio, Beijing, China) followed by microscopical evaluation.

### Anti-M3R autoantibody ELISA

M3R peptides as well as corresponding control peptides were dissolved in 0.5M Na_2_CO_3_ buffer (10 mg/ml) and 100 μl aliquots were distributed in wells of Costar EIA/RIA Plates (Corning Incorporated, NY, USA). After adsorption over night at 4°C, wells were rinsed and blocked with 3% BSA in PBS with 0.05% Tween-20 (PBS-T) for 1h at 37°C. Serum samples were 1:200 diluted in blocking buffer and incubated in peptide coated wells overnight at 4°C. After washing 50μlof peroxidase conjugated goat anti-human IgG antibodies (Sigma, USA) diluted 1:25000 in PBS-T supplemented with 0.5% BSA was added to each well. After incubation for 1 hour at room temperature, unbound antibodies were removed and bound antibodies were detected by using tetramethylbenzidine (Solarbio, Beijing, China) as the substrate for 15 minutes. The reaction was stopped by adding 50 μl of 2M sulphuric acid to each well. Optical density (OD) was measured at 450 nm by an ELISA spectrophotometer (ThermoMultiskan Mk3, Thermo,USA).

To determine human autoantibodies directed against the biotin conjugated M3R peptides biotin-2ndECL, biotin-c2ndECL, streptavidin solutions (50μl/well at 20μg/ml) in 0.5M Na_2_CO_3_ buffer (pH 9.6) were absorbed onto the Costar plates overnight at 4°C and subsequently blocked with 3% BSA in PBS-T for 1h at 37°C. After washing the plates with PBS-T, the biotin-2ndECL or biotin-c2ndECL solution (50μl/well at 20μg/ml) in PBS-T with 0.5% BSA were added onto the ELISA plates and incubated for 1.5hour at room temperature. Then autoantibodies against M3R peptides in the serum of patients and controls were determined as described above.

The titer of autoantibodies against the 2ndEL of M3R were expressed as arbitrary units (AU) and calculated according to the following formula: AU = (sample(OD^M3R peptide^—OD^non-specific control^)—negative control(OD^M3R peptide^—OD^non-specific control^))/ (positive control(OD^M3R peptide^—OD^non-specific control^) —negative control(OD^M3R peptide^—OD^non-specific control^)) ×100. Since there is no standardized positive control for anti-M3R peptide autoantibodies, we selected a sample which reacted positive in all 6 ELISA test systems as positive control, while a healthy control which scored negative in all 6 ELISA test variants served as negative control. The specificity of the positive signal was verified by using competitive ELISA using 2ndEL or c2ndEL as competitive peptides (S1 Fig).

To determine the murine IgG or its subclasses, IgG1/IgG2a/IgG2b/IgG3 against M3R peptides including N_ter_, 1stCEL, 2ndECL, c2ndECL, mouse sera were analyzed by ELISA according to the protocol described above with the modification of replacing the goat anti-human IgG antibody with goat anti-mouse IgG antibody (Sigma, USA) or goat anti-mouse IgG1/IgG2a/IgG2b/IgG3 antibodies (Santa Cruz, USA).

### Isolation of mouse salivary gland cells and Flow cytometry analysis

Mouse salivary gland cells were isolated from submandibular salivary glands as described [24], with minor modification. Briefly, murine submandibular salivary glands were mechanically chopped into a homogenous pulp and then digested by Collagenase IV and hyaluronidase (Sangon Biotech Shanghai, China). After washing, cell suspensions were passed through a 100 μm pore-size filter. To detect the IgG binding, one million primary murine salivary gland cells were washed three times with staining buffer (0.1% BSA in phosphate-buffered saline) and then incubated with 1:200 diluted mouse serum for 30 min at 4 degrees. After washing, cells were incubated in 100 μl staining buffer containing 0.1 μg Alexa Fluor 568 conjugated goat-anti-mouse IgG (Life technologies, USA) for 30 min at 4 degrees and finally suspended in 500 ul staining buffer containing 1% PFA. Bound murine IgG was quantified using a FACS Calibur Flow Cytometer (BD Biosciences)

### Statistical analysis

Quantitative data in normal distribution were compared using t-test, otherwise non-parametric test (Mann-Whitney U-test) was used for analysis. Frequency data were compared using Fisher's exact test. *P*<0.05 was considered to be statistically significant.

## Results

### Immunization with modified peptides induce a strong antibody response against peptides of the second extracellular loop of M3R in mice

**Fig 1:**
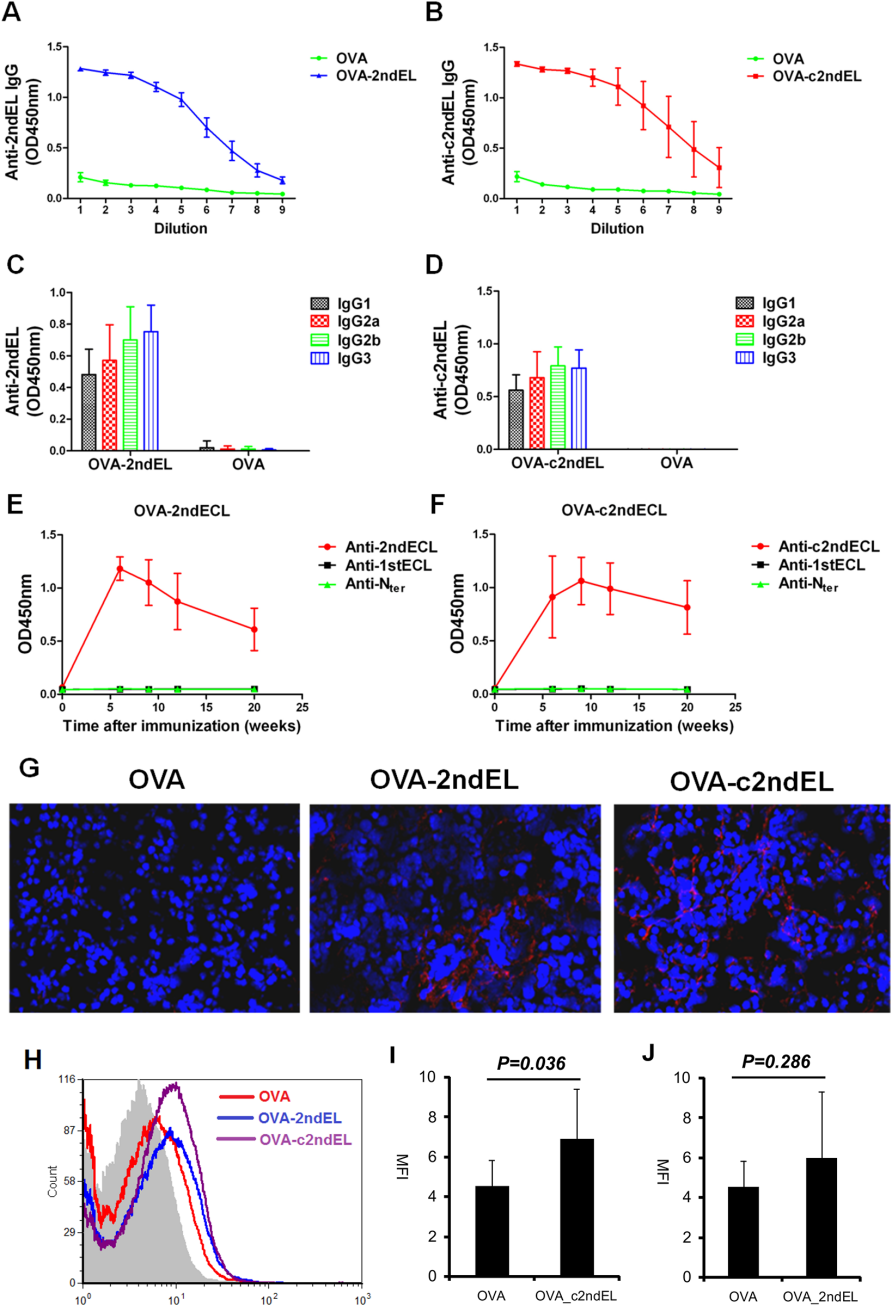
Induction of autoantibodies against peptides of the second extracellular loop of M3R in mice. Mice were immunized with the M3R-peptides OVA_2ndEL (A, C, E), OVA_2ndEL (B, D, F), or with the OVA peptide control. Antibodies in sera of immunized mice were tested for their binding to immobilized peptides. The titer of total IgG against 2ndEL (A, n = 4 per group) and c2ndEL(B, n = 4 per group) was determined in serum (the dilution factor as depicted) of mice 20weeks after immunization. The subclass of bound IgG, including IgG1, IgG2a, IgG2b and IgG3 against 2ndEL (C) and c2ndEL(D) was determined in samples analyzed in parallel. E and F represent time kinetics of autoantibody production against different peptides of M3R including N terminal (N_ter_), the first extracellular loop (1stEL) and the second extracellular loop (2ndEL or c2ndEL) in E) mice immunized with OVA_2ndEL peptide (n = 23)and controls (n = 15) or F) mice immunized with OVA_c2ndEL peptide (n = 8) and control (8). (G) Representative picture showing the IgG-deposition on salivary gland tissue of mice immunized with OVA-2ndEL, OVA-c2ndEL or OVA peptides. The IgG binding was detected by using direct immunofluorescence using DyLightTM649 Goat-anti-Mouse IgG antibody. (H) Representative FACS histogram showing the binding of IgG from OVA, OVA_2ndEL or OVA_c2ndEL immunized mice onto the primary murine salivary gland cells. Primary murine salivary gland cells were incubated with 1:200 diluted sera from OVA (n = 8), OVA_2ndEL (n = 8) or OVA_c2ndEL (n = 8). The bound IgG were detected by Alexa Fluor 568 conjugated goat-anti-mouse IgG. (I and J) Quantified IgG bound onto primary murine gland cells. All data are presented as mean±SD. *P* values were calculated using Student's t test.

As cyclic peptides are believed to mimic the conformational epitopes, we induced mice to produce autoantibodies against the linear as well as the cyclic peptides of the second extracellular loop of M3R. In order to break the immune tolerance for self-antigen peptides, we immunized the mice with OVA_323–339 peptide conjugated to the respective M3R peptides, resulting in OVA-2ndEL, OVA-c2ndEL, or OVA control peptides. Since the OVA-323-339 peptide is a strong T cells epitope which can be recognized by Balb/c strain carrying H2-d [25], the mice were expected to produce autoantibodies against M3R peptides via the mechanism of linked recognition [26]. As predicted, mice immunized with OVA-2ndEL peptides produced high amount of autoantibodies against the 2ndEL peptides, while mice immunized with OVA control peptide did not (Fig 1A). Similarly, mice immunized with OVA-c2ndEL produced large amount of autoantibodies against the cyclic c2ndEL peptide (Fig 1B). We then further determined the IgG subclasses of the autoantibodies against the M3R peptides. As shown in Fig 1C and 1D, all four IgG subclasses including IgG1, IgG2a, IgG2b and IgG3 were generated in mice immunized with OVA-2ndEL or OVA-c2ndEL peptide.

Since the OVA_323–339 peptide represents the T cell epitope within the immunized antigen, the mice were supposed to produce antibodies against the conjugated peptides of M3R but not antibodies against peptides of other M3R domains. To verify this, we determined the autoantibody production against the peptides of the N-terminal domain, the 1stEL and the 2ndEL of the M3R. As shown in Fig 1E and 1F, all mice immunized with OVA-2ndEL or OVA-c2ndEL produced autoantibodies reactive to the second extracellular loop of M3R, which lasted even 20 week after the immunization. However, the immunized mice did not produce autoantibodies reactive to the peptides of N-terminal domain or the first extracellular loop of M3R.

We next examined whether the autoantibodies generated in the immunized mice could bind to exocrine glands. By using direct immunofluorescence staining we observed IgG deposition on salivary acinar cells in mice immunized with OVA-2ndEL or with OVA -c2ndEL while samples from mice immunized with OVA control peptide were negative (Fig 1G). To further quantify this binding, primary salivary gland cells were prepared from healthy mice and incubated with sera of differentially immunized mice. Bound IgG was detected by goat-anti-mouse IgG staining and quantified by flow cytometry. As shown in Fig 1H and 1I, sera from OVA_c2ndEL peptide immunized mice displayed a significant higher binding to primary salivary gland cells than sera derived from OVA control peptide immunized mice (*P* = 0.036). In contrast, the binding of the sera from OVA_2ndEL peptide immunized mice was not significantly higher than that of the control sera (Fig 1J). These results indicate that autoantibodies raised against the cyclic peptides of the second extracellular loop of M3R are able to bind to salivary gland cells.

### Immune reactivity against peptides of the second extracellular loop of M3R is not associated with pSS-like disease

**Fig 2:**
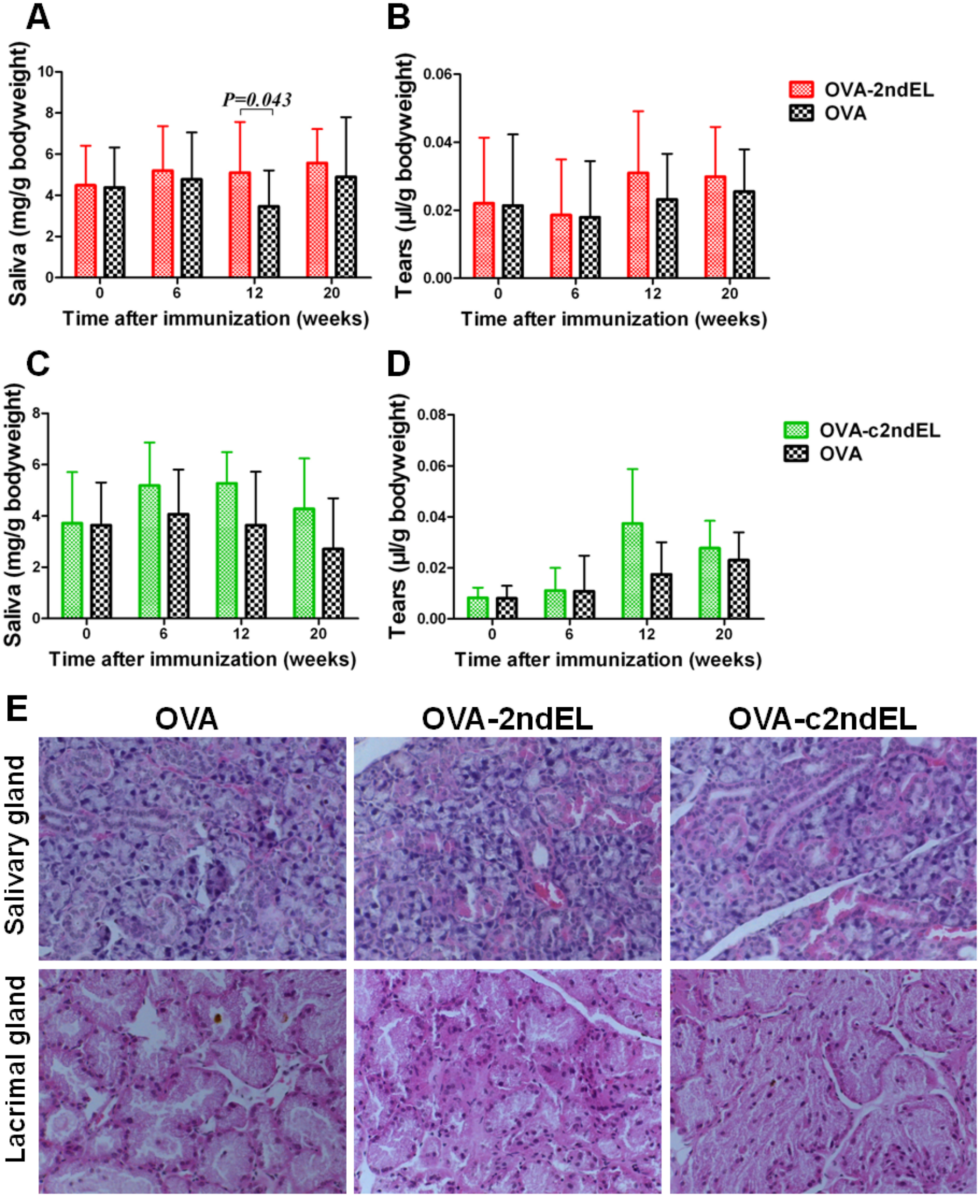
Function and histology of exocrine glands of peptide-immunized mice. The secretion of saliva (A, C) and tears (B, D) of mice immunized with OVA-2ndEL peptide (A, B) (n = 23), OVA-c2ndEL peptides (n = 8) (C, D)or OVA control peptide (A (n = 15), and D (n = 8)). (E). Representative H&E-stained sections of salivary and lacrimal glands isolated from the mice immunized with OVA-2ndEL, OVA-c2ndEL, or OVA peptide. Eight mice from each group were histologically evaluated, and no lymphocytic focus was observed. All data are presented as mean±SD. *P* values were calculated by Student's t test.

The specific induction of the tissue-binding autoantibodies against the second extracellular loop of M3R allowed us to investigate the role of those autoantibodies in the pathogenesis of pSS. Consequently, we evaluated the function of the exocrine glands by determining the secretion of saliva and tears at 0, 6, 12 and 20 weeks postimmunization. As compared to animals immunized with the OVA control peptide, neither saliva nor tear secretion was decreased at any time point in mice after immunization with OVA-2ndECL or OVA-c2ndECL peptides (Fig 2A–2D). To the contrary, the only significant difference between these groups was observed in the OVA-2ndEL immunized mice at 12 weeks post-immunization, where the these animals produced slightly more saliva than the corresponding controls(5.10±2.46 mg/g *vs* 3.28±1.74mg/g, *P* = 0.043), (Fig 2A). Taken together, the induction of the tissue binding autoantibodies against linear or cyclic peptides of the 2ndEL of M3R did not result in a decrease in the secretion of saliva or tears.

Most pSS patients are characterized with focal lymphoid infiltration in the minor salivary gland. We, therefore, analyzed the development of such lymphocytic infiltrates in the exocrine glands of immunized mice as a second disease read-out. Submandibular and lacrimal glands collected from mice at 20^th^ week post-immunization were evaluated for the lymphocytic infiltration by histological staining. As shown in the Fig 2E, no focal lymphoid infiltration was found in any exocrine glands. In addition, there was also no other histological abnormality in the OVA-2ndEL or OVA-c2ndEL immunized mice as compared with controls.

### Transfer of the autoantibodies against the peptides of the second extracellular loop of M3R does not impair the function of the exocrine glands

**Fig 3:**
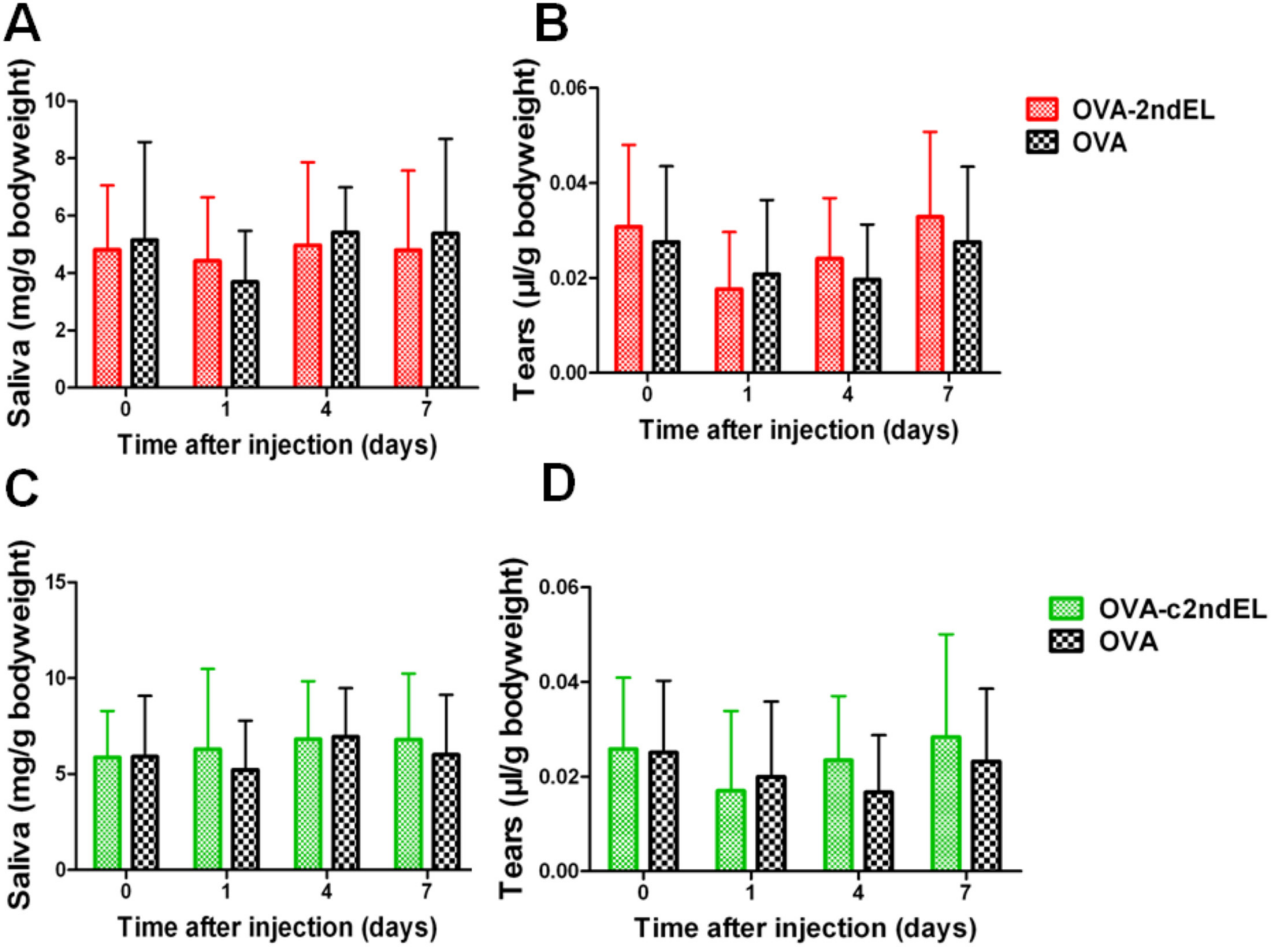
Transfer of IgG derived from peptide-immunized mice does not induce impairment of tear and salivary secretion in healthy animals. The production of saliva (A, C) and tears (B, D) of the mice injected 400 μg IgG isolated from mice immunized with OVA-2ndEL (A, B) (n = 9) or OVA-c2ndEL (C, D) (n = 13) or 400 μg IgG from mice immunized with OVA peptide (A, B; n = 9 and B, C; n = 16; respectively).

To further determine the potential pathogenicity of autoantibodies to peptides of the second extracellular loop of M3R, we isolated the total IgG from the serum of mice immunized with OVA-2ndECL, OVA-c2ndECL or OVA peptides. After injection of IgG fractions into healthy Balb/c mice, saliva and tears secretion was determined at days 0, 1, 4, and 7 after IgG transfer. As shown in Fig 3A and 3B, transfer of IgG isolated from OVA-2ndECL immunized mice did not provoke relevant differences in either saliva or tear secretion of the recipient mice during the time period analyzed as compared animal receiving control antibodies from OVA immunized animals. Comparable results were obtained in experiments where IgG derived from OVA-2ndECL immunized mice was used (Fig 3C and 3D). These results suggest that transfer of antoantibodies against the peptides of the second extracellular loop of M3R had no effect on the secretion of saliva and tear in recipient mice.

### Autoantibodies against peptides of the second extracellular loop of M3R are not suitable as biomarker in pSS patients

**Fig 4:**
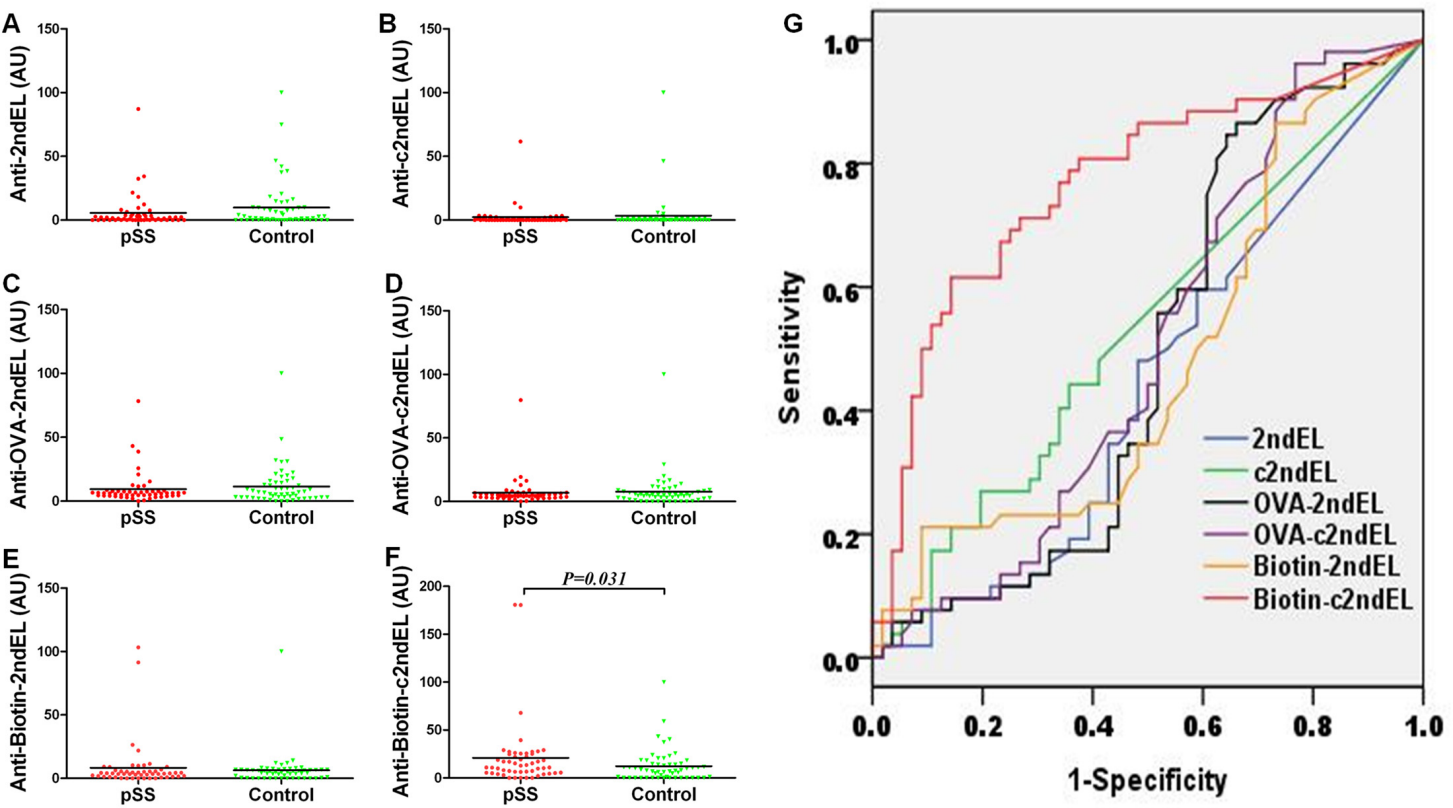
Autoantibodies directed against peptides of the second extracellular loop of the M3R in pSS patients and healthy controls. Sera derived from pSS patients and healthy controls were collected and diluted 1:200 before use. The ELISA was performed for three linear peptides 2ndEL (A), OVA-2ndEL(C) and biotin-2ndEL(E), and three cyclic peptides c2ndEL(B), OVA-c2ndEL(D) and biotin-c2ndEL(F) of the second extracellular loop of M3R. The amount bound antibodies was expressed as arbitrary units (AU). G. Receiver operating characteristics curves for the six peptides were made using SPSS 17 software to determine sensitivity and specificity of the tested peptides. The *P* value was calculated using the Mann-Whitney U-test.

Although autoantibodies against the peptides of the second extracellular loop of M3R are apparently not involved in pSS pathogenesis, we next investigated if they could be used as biomarker for pSS in patients. We first detected the autoantibodies against the unmodified peptides 2ndEL, c2ndEL using SGSG peptide as a control. The mean levels of the anti-2ndEL IgG in pSS patients (5.6±13.7AU) was not significantly different compared to those in controls (9.8±18.8AU) (Fig 4A). Furthermore, reactivity of patient IgG to the cyclic peptide, c2ndEL(2.3±8.7AU) differed also not significantly from antibody titers observed in healthy controls (3.4±14.6AU) (Fig 4B). By defining a positive test result as an AU >mean+2SD of the values of healthy controls, the estimated frequency of positive sample in patients were 1.92% (anti-2ndEL) and 3.57% (anti-c2ndEL). However, frequencies were not significantly different from those observed in controls (Table 2).

**Table 2:** Summary of the autoantibodies against the linear or cyclic peptides of the second extracellular loop of M3R in healthy control and pSS patients.

Peptides	pSS		Controls	
	AU (Mean±SD)	% of positive samples	AU (Mean±SD)	% of positive samples
**2ndEL**	5.6±13.7	1.92%	9.8±18.8	3.57%
**c2ndEL**	2.3±8.7	1.92%	3.4±14.6	3.57%
**OVA-2ndEL**	9.4±12.6	3.84%	11.4±15.5	3.57%
**OVA-c2ndEL**	6.8±11.0	1.92%	7.7±13.8	1.79%
**Biotin-2ndEL**	8.3±18.7	3.84%	6.3±13.2	1.79%
**Biotin-c2ndEL**	20.9±34.5*	5.77%	12.3±17.1	3.57%

**Note:** The positive sample was defined as a sample with AU value>mean+2SD of the healthy subjects.

* *P*<0.05 (compared with controls), Mann-Whitney U-test.

Binding of antibodies to immobilized short peptides like those used in this study may be limited by steric hindrance. To circumvent this problem, we determined the immune reactivity of patient sera towards OVA peptide conjugatedM3R peptides (OVA-2ndECL and OVA-c2ndECL) or the corresponding OVA control peptide. As shown in Fig 4C, there was no differences in the titer of anti-OVA-2ndEL IgG between pSS patients (9.4±12.6 AU) and controls (11.4±15.5 AU). Also, no significant difference was observed in autoantibodies against the cyclic OVA-c2ndEL between pSS patients (6.8±11.0 AU) and controls (7.7±13.8AU) (Fig 4D). In addition, none of the frequencies of positive samples of anti-OVA-2ndEL IgG or anti-OVA-c2ndEL IgG was significantly different between pSS patients and healthy controls (Table 2).

To overcome the disadvantages of associated with an unspecific coating of peptides to surfaces, we immobilized strepavidin onto the plate to capture biotin-conjugated M3R peptides biotin-2ndECL and biotin-c2ndECL. However, even with this new approach, anti-biotin-2ndEL IgG titer in pSS patients (8.3±18.7 AU) appeared not to be elevated as compared to those in healthy controls (6.3±13.2 AU) (Fig 4E). The frequency of the anti-biotin-2ndEL IgG positive samples in pSS patients was also similar to that in controls (Table 2). It should be mentioned that a significant difference was observed in the titer of autoantibodies against the biotin modified cyclic peptide, where the titer of the anti-biotin-c2ndEL IgG in pSS patients was slightly higher than that in controls (20.9±34.5 AU *vs* 12.3±17.1 AU, *P* = 0.031). However, no significant difference was observed in the frequencies of the anti-biotin-c2ndEL IgG positive samples between pSS patients (5.77%) and controls (3.57%) (Fig 4F and Table 2).

To determine sensitivity and specificity of each peptide, Receiver operating characteristics (ROC) graphs were made and the areas under the curves (AUC) were calculated (Fig 4G). None of the AUC reached statistically significance, suggesting that neither sensitivity nor specificity of any of the above six peptide-based ELISA approaches represents a reliable tool for the discrimination between healthy subjects and pSS patients.

## Discussion

In the present study we raised autoantibodies in mice by immunization withM3R peptides derived from the second extracellular loop conjugated to OVA_323–339, OVA_2ndEL and OVA_c2ndEL. Breaking tolerance is based here on linked recognition in which B cell recognizing a certain epitope is activated by helper T cells recognizing a second epitope within the same antigen [26]. Since the T cell epitopes and B cell epitopes on a certain antigen are frequently not overlapped, linked recognition is a common phenomenon for antibody production. Due to the immune tolerance, induction of some autoantibodies requires immunization with modified antoantigens, e.g. recombinant autoantigens bearing a GST-tag, coupling of autoantigens to OVA, or heterologous autoantigens [27,28]. With these modifications, non-self T cell epitopes are introduced and help B cells to produce autoantibodies via linked recognition. However, immunization with autoantigens conjugated to a synthesized T cell epitope has not yet been reported. To our knowledge, this study is the first one to overcome the immune tolerance by immunizing a synthesized peptide composed of a self B cell epitope and a non-self T cell eiptope. This novel approach can be used to generate autoantibodies *in vivo*, particularly for autoantibodies against linear epitopes.

Surprisingly, the induced autoantibodies directed against cyclic peptides of the second extracellular loop of M3R are capable in binding to the exocrine glands but do not impair the function of gland secretion, suggesting that they are not pathogenic. This is the first *in vivo* study investigating the pathogenicity of autoantibodies against the peptides of M3R. Although there have been some *in vitro* evidences showing that autoantibodies against the peptides of the second extracellular loop of M3R might be pathogenic [12,13,29], our findings *in vivo* strongly argue against this hypothesis. Our results are further substantiated by our observation that in humans such autoantibodies are expressed in some healthy control subjects which, of course, have no clinical symptoms. However, these results do not necessarily exclude M3R as a pathogenic autoantigens of pSS. First of all, the synthesized peptides of the second extracellular loop of M3R, either in linear or cyclic form, might insufficiently mimic the conformational epitopes of the M3R. Thus, autoantibodies targeting these peptides may be unable to block the function of M3R. Second, since autoantibodies against M3R recognize multiple epitopes of the protein [30], it cannot be excluded that pathogenic epitopes are located outside of the second extracellular loop of the receptor. Finally, a T cell-dependent but not autoantibody-dependent mechanism in pSS pathogenesis has been shown by Ilzuka et al. in a mouse model where mice were immunized with M3R peptides [10], indicating that the cellular rather than the humoral immune response against M3R play the predominant role in the pathogenesis of pSS.

In the present study, we determined the autoantibodies against the linear and cyclic peptides of the second extracellular loop of M3R in pSS patients and healthy controls using different peptide-based ELISA approaches. However, none of our settings enabled a significant discrimination between pSS patients and healthy control subjects. Since murine autoantibodies are detectable in this setting, the low prevalence of the human autoantibodies against the peptides of second extracellular loop of M3R is unlikely due to the technique reasons. Therefore, these results suggest that the peptide-based ELISA used here is not suitable tool to detect the autoantibodies against M3R in pSS patients. This finding is particularly consistent with a previous report by Roescher *et al*. demonstrating that peptide-based ELISAs were neither sensitive nor specific enough for detecting autoantbodies against M3R in pSS [20].

In summary, we have induced an autoantibody immune response against peptides of the second extracellular loop of M3R in mice by immunization with a synthesized peptide composed of a self B cell epitope and a non-self T cell epitope. Using this *in vivo* approach, we put previous *in vitro* findings on a disease promoting role of autoantibodies against the peptides of the second extracellular loop of M3R in pSS into perspective. The role of immune response against M3R in the pathogenesis of pSS needs to be further elucidated. Furthermore, the prevalence of those autoantibodies in pSS patients is rather low and does not differ from that in healthy controls, suggesting that they are not sufficient to serve as reliable biomarkers for the disease.

## Supporting Information

S1 Fig Competitive ELISA curves for detecting autoantibodies against M3R peptides. (PDF)
