# Supplementary material for: Autoantibodies against the Second Extracellular Loop of M3R Do neither Induce nor Indicate Primary Sjögren’s Syndrome
Source: PLoS One. 2016 Feb 22;11(2):e0149485. doi: 10.1371/journal.pone.0149485 (PMC4765836; doi:10.1371/journal.pone.0149485)
Supplement: S1 Fig — (PDF) [file pone.0149485.s001.pdf]

Supporting information

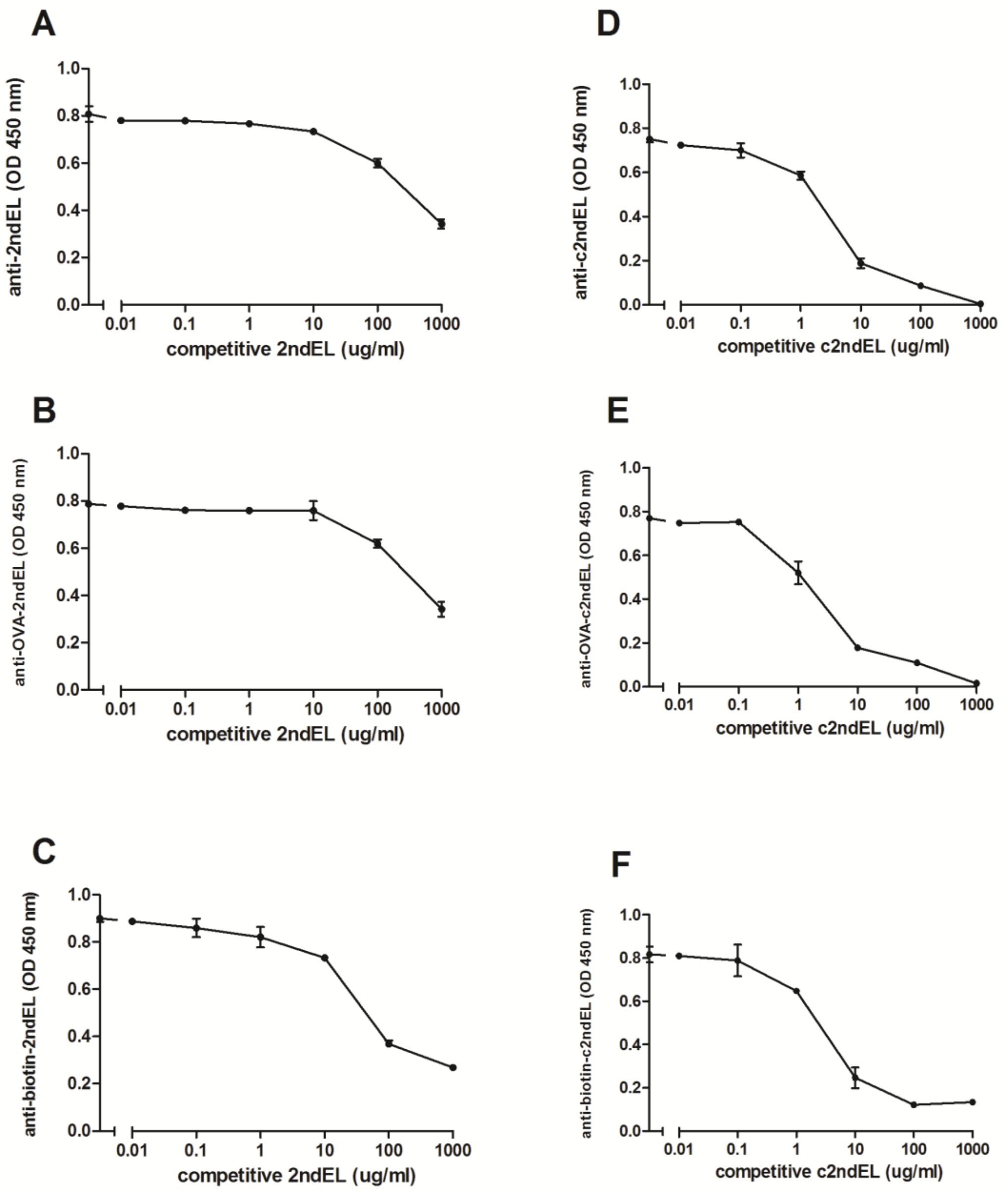

S1 Fig. Competitive ELISA curves for detecting autoantibodies against M3R peptides. Using 2ndEL as the competitive peptide, the specificity of the autoantibodies against 2ndEL was determined by using 2ndEL (A), OVA-2ndEL (B) or bio-2ndEL (C) as the coated antigen. Using c2ndEL as the competitive peptide, the specificity of the autoantibodies against c2ndEL was determined by using c2ndEL (D), OVA-c2ndEL (E) or coating bio-c2ndEL as the coated antigen (F) .
